# Role of the JAK2/STAT3 pathway on infection of *Francisella novicida*

**DOI:** 10.1371/journal.pone.0310120

**Published:** 2024-09-10

**Authors:** Sonoko Matsumoto, Takashi Shimizu, Akihiko Uda, Kenta Watanabe, Masahisa Watarai

**Affiliations:** 1 Laboratory of Veterinary Public Health, Joint Faculty of Veterinary Medicine, Yamaguchi University, Yamaguchi, Japan; 2 One Welfare Education and Research Center, Joint Faculty of Veterinary Medicine, Yamaguchi University, Yamaguchi, Japan; 3 Department of Veterinary Science, National Institute of Infectious Diseases, Shinjuku, Tokyo, Japan; University of Limpopo, SOUTH AFRICA

## Abstract

*Francisella tularensis* is a causative agent of the zoonotic disease tularemia, and is highly pathogenic to humans. The pathogenicity of this bacterium is largely attributed to intracellular growth in host cells. Although several bacterial factors important for the intracellular growth have been elucidated, including the type VI secretion system, the host factors involved in the intracellular growth of *F*. *tularensis* are largely unknown. To identify the host factors important for *F*. *tularensis* infection, 368 compounds were screened for the negative regulation of *F*. *tularensis* subsp. *novicida* (*F*. *novicida*) infection. Consequently, 56 inhibitors were isolated that decreased *F*. *novicida* infection. Among those inhibitors, we focused on cucurbitacin I, an inhibitor of the JAK2/ STAT3 pathway. Cucurbitacin I and another JAK2/STAT3 inhibitor, Stattic, decreased the intracellular bacterial number of *F*. *novicida*. However, these inhibitors failed to affect the cell attachment or the intrasaccular proliferation of *F*. *novicida*. In addition, treatment with these inhibitors destabilized actin filaments. These results suggest that the JAK2/STAT3 pathway plays an important role in internalization of *F*. *novicida* into host cells through mechanisms involving actin dynamics, such as phagocytosis.

## Introduction

*Francisella tularensis* are gram-negative bacteria that can be distinguished into four subspecies; *F*. *tularensis* subsp. *tularensis* (type A), *F*. *tularensis* subsp. *holarctica* (type B), *F*. *tularensis* subsp. *mediasiatica*, and *F*. *tularensis* subsp. *novicida* (*F*. *novicida*). Among them type A and type B *F*. *tularensis* have been reported to be pathogenic to humans [1]. *F*. *tularensis* is the zoonotic causative agent of tularemia, which occurs in Northern Hemisphere countries [1]. As few as 10 bacteria of type A *F*. *tularensis* inhaled through aerosols develop tularemia in humans [2]. Consequently, because of the high infectivity of *F*. *tularensis*, the Centers for Disease Control and Prevention (USA) is concerned for misuse of *F*. *tularensis* in terrorism and has classified this bacterium as a category A bioterrorism agent [3]. *F*. *novicida* is highly related to type A *F*. *tularensis* genetically but exhibits low pathogenicity in humans [1]. However, *F*. *novicida* is pathogenic to mice and is a commensal intracellular pathogen that replicates in both human and mouse macrophages in the same way as seen with type A *F*. *tularensis* [4]. Thus *F*. *novicida* is thought to have considerable homology with type A *F*. *tularensis* and is often used as a practical surrogate of type A *F*. *tularensis* [5]. Although there are many reports on virulence factors of *Francisella*, including the presence of a type VI secretion system [6], the host factors important for the infection of the bacteria are largely unknown.

*Francisella* are ingested through the pseudopodial loop of macrophages and incorporated into spacious vacuoles that have endosomal markers [7, 8]. The bacterium then escapes from the phagosomal membrane and replicates in the cytoplasm [9]. In the late stages of infection, *Francisella* bacteria re-enter the autophagosome [10] and acquire amino acids from degraded proteins and replicate [10]. Cytoplasmic bacteria with defective or damaged replication are trapped in *Francisella*-containing vacuoles, which are lysosome-associated membrane protein 1 (LAMP-1)-positive autophagosomes and are degraded via the ubiquitin-SQSTM1-LC3 pathway [11, 12].

The JAK2/STAT3 pathway is involved in various biological processes such as immunity, cell division, cell death, and tumor formation [13]. Activation of the JAK2/STAT3 pathway occurs via the binding of the extracellular domain of specific intracellular receptors (RTKs) to hormones (e.g., prolactin), growth factors (e.g., epidermal growth factor, EGF), and cytokines (e.g., the interleukin-6, IL-6, family). JAK2 mediates signaling through several cytokine receptors, including IL-6 and IFN-γ. The interaction between ligands and receptors induces dimerization of the receptor subunits. The close proximity of JAK2 noncovalently bound to the intracellular domain of the receptor causes autophosphorylation of JAK2 and stimulates the kinase activity [14, 15]. When the JAK2 protein is phosphorylated, tyrosine residues in the intracellular domain of the receptor are phosphorylated by the activated JAK2 kinase domain, creating a docking site for STAT3 within the SH2 domains of the receptor. This allows cytoplasmic STAT3 protein to bind to the phosphorylated tyrosine residues on the receptor. STAT3 is then phosphorylated by JAK2, dimerizes, dissociates from the receptor, and moves to the nucleus. The phosphorylated STAT3 dimer binds to specific DNA sequences, inducing transcription of target genes, including Cyclin D1, cMyc, Bclx1, bcl-2, MCL-1, and P53, leading to cell proliferation, differentiation, apoptosis, and immune regulation [15]. Furthermore, JAK2/STAT3 signaling can interact with other pathways, including the MAPK/ERK and PI3K/ACT/mTOR signaling pathways, to activate specific cellular responses [16].

In this study, we sought to identify the host factors important for *Francisella* infection and screened 368 compounds for those that inhibited *F*. *novicida* infection. Consequently, we focused on cucurbitacin I, an inhibitor of JAK2/STAT3 [17], and investigated the effect of JAK2/STAT3 pathway on *F*. *novicida* infection.

## Materials and methods

All experiments were conducted in accordance with the institutional biosecurity guidelines and were approved by Yamaguchi University.

### Inhibitor library and inhibitors

The inhibitor compound library was obtained from Molecular Profiling Committee, Grant-in-Aid for Transformative Research Areas “Advanced Animal Model Support (AdAMS)” from The Ministry of Education, Culture, Sports, Science, and Technology, Japan (JSPS KAKENHI Grant Number JP 22H04922). Cucurbitacin I (Merck, Darmstadt, Germany) and Stattic (Merck) were dissolved in dimethyl sulfoxide (DMSO) at 2 mM and then diluted to 200, 20, and 2 μM. The same amount of the inhibitors and DMSO control were added to culture medium at a final concentration of 10, 1, 0.1, and 0.01 μM.

### Cell culture

The mouse monocyte-macrophage J774.1 cell line was cultured at 37°C under 5% CO_2_ in Roswell Park Memorial Institute (RPMI) 1640 medium (Thermo Fisher, Waltham, MA) supplemented with 10% fetal bovine serum (Thermo Fisher).

### Bacteria strains and culture conditions

*F*. *novicida* ATCC 15482 strain was cultured aerobically at 37°C with brain heart infusion broth (Becton, Dickinson and Company, Franklin Lakes, NJ) supplemented with cysteine (BHIc), BHIc plates containing 1.5% Agar (Wako Laboratory Chemicals, Osaka, Japan) [18], or chemically defined medium (CDM) [19]. Green-Fluorescent protein (GFP)-expressing *F*. *novicida* was cultured with BHIc containing 2.5 μg/mL chloramphenicol [20]. *Escherichia coli* ATCC15482 strain was cultured aerobically at 37°C in Luria–Bertani medium (LB) (Nacalai Tesque, Kyoto, Japan) or LB plates containing 1.5% agar. Bacterial concentrations were adjusted based on their optical density at 595 nm.

### Screening of inhibitors

J774.1 cells (25 × 10^4^ cells/mL) were seeded at 100 μL/well in a 96-well plate or 500 μL/well in a 24-well plate and cultured overnight. Cells were treated with 10 or 1 μM of inhibitors for 2 h; DMSO was used as the control. After treatment, cells were infected with GFP-expressing *F*. *novicida* at multiplicity of infection (MOI) of 1 and incubated for 24 h. Cells were then washed three times with phosphate-buffered saline (PBS) and fluorescence intensity was measured using plate reader 2030 ARVO X4 (Perlin Elmer, Waltham, MA). An intensity at 4,000 lower than that of the DMSO control showed inhibition of infection, whereas an intensity at 5000 higher than that of the control showed enhancement of infection (S1 Table).

### Inhibitor treatment before infection

J774.1 cells (25 × 10^4^ cells/mL) were seeded at 100 μL/well in a 96-well plate or 500 μL/well in a 24-well plate and cultured overnight. Cells were treated with indicated concentration of cucurbitacin I or Stattic for 2 h; DMSO was used as the control. After treatment, cells were infected with *F*. *novicida* at an MOI of 1. Plates were centrifuged for 10 min at 300 × *g* and incubated for 1 h at 37°C. Cells were cultured in culture medium containing inhibitors, and 50 μg/mL of gentamycin added for 1 h to kill extracellular bacteria. Cells were then washed three times with PBS and cultured in medium containing inhibitors at 37°C.

### Inhibitor treatment after infection

J774.1 cells (25 × 10^4^ cells/mL) were seeded at 100 μL/well in a 96-well plate or 500 μL/well in a 24-well plate and cultured overnight. Cells were infected with *F*. *novicida* at an MOI of 1. Plates were centrifuged for 10 min at 300 × *g* and incubated for 1 h at 37°C. Cells were treated with 50 μg/mL of gentamycin to kill extracellular bacteria. Cells were washed three times with culture medium and treated with indicated concentrations of cucurbitacin I or Stattic.

### Colony forming units (CFU)

J774.1 cells (25 × 10^4^ cells/mL) were seeded at 100 μL/well in a 96-well plate and cultured overnight. After inhibitor treatment and infection described above, cells were washed three times with PB and then disrupted with 0.1% Triton-X in CDM for 1 min and 900 μL of CDM was immediately added. Samples were diluted with CDM and cultured on a BHIc plate overnight, and the number of colonies were counted.

### Laser scanning confocal microscopy

J774.1 cells (25 × 10^4^ cells/mL) were seeded at 500 μL/well in a 24-well plate with 120-mm glass coverslips (Matsunami, Osaka, Japan) and cultured overnight. After treatment with inhibitors and infection with GFP-expressing *F*. *novicida* as described above, cells were washed three times with PBS and fixed with 4% paraformaldehyde in PBS for 30 min at room temperature. Images of the cells were obtained using FluoView FV100 confocal laser scanning microscope (Olympus, Tokyo, Japan).

### Measurement of phagocytotic activity

The activity of phagocytosis against *E*. *coli* was measured as previously described with slight modification [21, 22]. J774.1 cells (25 × 10^4^ cells/mL) were seeded at 100 μL/well in a 96-well plate or 500 μL/well in a 24-well plate and cultured overnight. Cells were treated with the indicated concentration of cucurbitacin I, Stattic, or DMSO control for 2 h. After treatment, cells were infected with *E*. *coli* for 3 h and treated with gentamycin to kill extracellular bacteria. Cells were then washed three times with PBS and disrupted with 0.1% Triton-X in PBS for 1 min followed by immediate addition of 900 μL PBS. CFU was counted as described above.

### Visualization of actin filaments

J774.1 cells (25 × 10^4^ cells/mL) were seeded at 500 μL/well in a 24-well plate with 12-mm glass coverslips and cultured overnight. After treatment of inhibitors and infection described above, cells were washed three times with PBS and fixed with 4% paraformaldehyde in PBS. After being washed three times with PBS, cells were permeabilized with 0.1% Triton-X in PBS for 5 min and washed three times with PBS. Cells were then blocked with 2% of bovine serum albumin in PBS for 1 h. and stained with 0.1 μM of phalloidin-rhodamine B isothiocyanate (P1951, Thermo Fisher) for 2 h. Cells were washed three times with PBS and observed with laser scanning confocal microscopy.

### Statistical analysis

Significant differences are determined by *P* < 0.05 or *P* < 0.01 using Student’s t-test or Dunnett’s test, or Tukey–Kramer method, and indicated with * and ** respectively.

## Results

### Screening of inhibitors

To identify host factors important for the infection of *F*. *novicida*, 368 inhibitor compounds were screened to identify those that inhibited the growth of *F*. *novicida*. J774.1 cells treated with inhibitors for 2 h were infected with GFP-expressing *F*. *novicida*, and cells with a lower fluorescence compared with that of untreated cells were selected (**S1 Table**). Consequently, 56 inhibitors were selected to inhibit the growth of *F*. *novicida* in J774.1 cells (Table 1). A number of the identified inhibitors have been demonstrated to possess antibiotic properties. Among the non-antibiotic inhibitors, three were found to be involved in the Jak-2/Stat3 pathway. Consequently, we considered that the Jak-2/Stat3 pathway was a key factor in *F*. *novicida* infection and focused on cucurbitacin I as a representative inhibitor of the JAK2/STAT3 pathway.

**Table 1:** Inhibitors negatively regulated the infection of *F*. *novicida*.

Compound	Category
5,15-DPP	STAT3
ABT-702	AK
Actinonin	aminopeptidase M
AG957	Bcr-abl
Amastatin	aminopeptidase A
AMD3100 octahydrochloride	CXCR4
Aminoglutethimide	aromatase
anisomycin	stress inducer
Bafilomycin A1	V-ATPase
BH3I-1	Bcl-XL
bortezomib	Proteasome
brefeldin A	golgi inhibitor
CA-074	cathepsin B
Cdk4 inhibitor	CDK
Chetomin	HIF
Cucurbitacin I	Jak-2
cyclopamine	Hedgehog
Cytochalasin D	actin filament
E-64d	calpain
Formestane	aromatase
gefitinib	EGFR
Glibenclamide	K channel
HA 14–1	Bcl-2
imatinib mesylate	Bcr-Abl/Kit
Jervine	Hedgehog
KT5823	PKG
Leptomycin B	CRM1
LFM-A13	Burton’s tyrosine kinase(BTK)
MG-132	proteasome
Mifepristone	progesterone receptor
Monensin	Na ionophore
Nifedipine	Ca channel
Nigericin	K ionophore
nilotinib	Bcr-Abl
NSC625987	CDK
NSC95397	Cdc25
Ouabain	Na/K ATPase
Pepstatin A	cathepsin D
Pifithrin-a (cyclic)	p53
PIM1 Inhibitor II	PIM
PKR inhibitor	PKR
PRIMA-1	p53 activator
radicicol	Hsp90
Rotenone	mitochondrial complex I
RS 102895	CCR2
Sanguinarine	Na/K/Mg ATPase
SB 328437	CCR3
sorafenib	Multi-kinases
sunitinib malate	Multi-kinases
temsirolimus	mTOR
Thapsigargin	Ca-ATPase
TOFA	acetyl-CoA carboxylase (ACC)
vorinostat	HDAC
WP1066	STAT3
YM155	Survivin
Z-GLF-CMK	cathepsin G

### Effect of inhibitors on *F*. *novicida* infection

**Fig 1:**
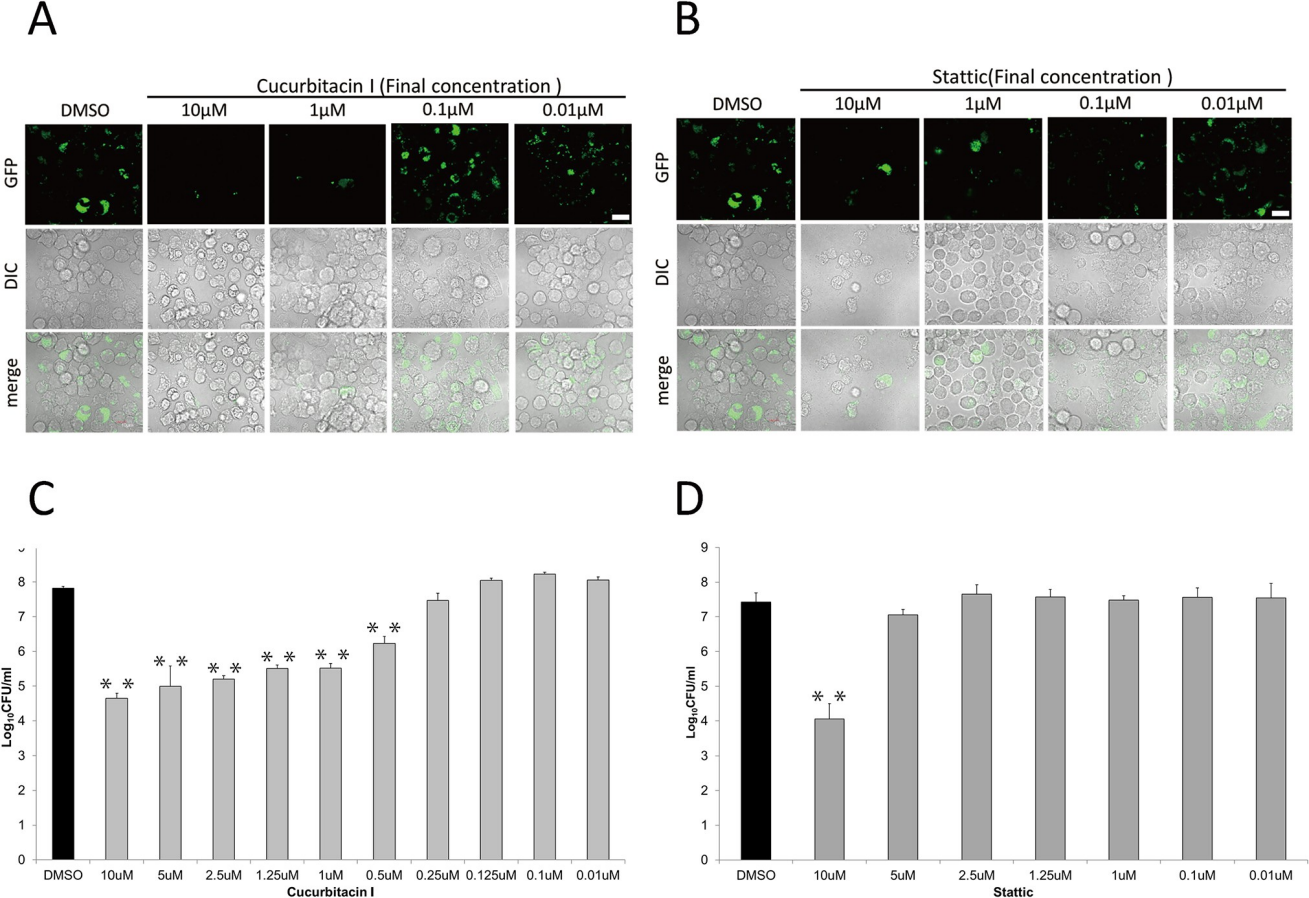
Suppression of *F*. *novicida* infection by JAK2/STAT3 inhibitors. J774.1 cells treated with 0.01 to 10 μM of cucurbitacin I (A, C) or Stattic (B, D) were infected with *F*. *novicida* (C, D) or GFP-expressing F. novicida (A, B). Cells were treated with gentamicin and incubated for 12 h and then observed with confocal microscopy (A, B), or the number of intracellular bacterial was counted (C, D). Data represent the average and standard deviation of three identical experiments. Differences were analyzed with multiple comparison (Dunnett’s test) and indicated by asterisks, **P < 0.01, *P < 0.05. Scale bar: 10μm.

To assess whether cucurbitacin I suppressed *F*. *novicida* infection, J774.1 cells treated with cucurbitacin I were infected them with GFP-expressing *F*. *novicida*, and intracellular bacteria were observed using confocal microscopy. Cucurbitacin I-treated J774.1 cells were also infected with *F*. *novicida*, and the number of intracellular bacteria was measured by colony counting. Cucurbitacin I treatment decreased the number of intracellular *F*. *novicida* (Fig 1A, 1C), indicating that the JAK2/STAT3 pathway is important for *F*. *novicida* infection. To confirm this, the effect of another JAK2/STAT3 pathway inhibitor Stattic was evaluated. Stattic treatment decreased the number of intracellular *F*. *novicida* as assessed by both microscopic observation and colony counting (Fig 1B, 1D). A concentration of 1 μM cucurbitacin and 10 μM of Stattic were significantly effective and were therefore used in subsequent experiments.

### Growth of *F*. *novicida* in BHIc

**Fig 2:**
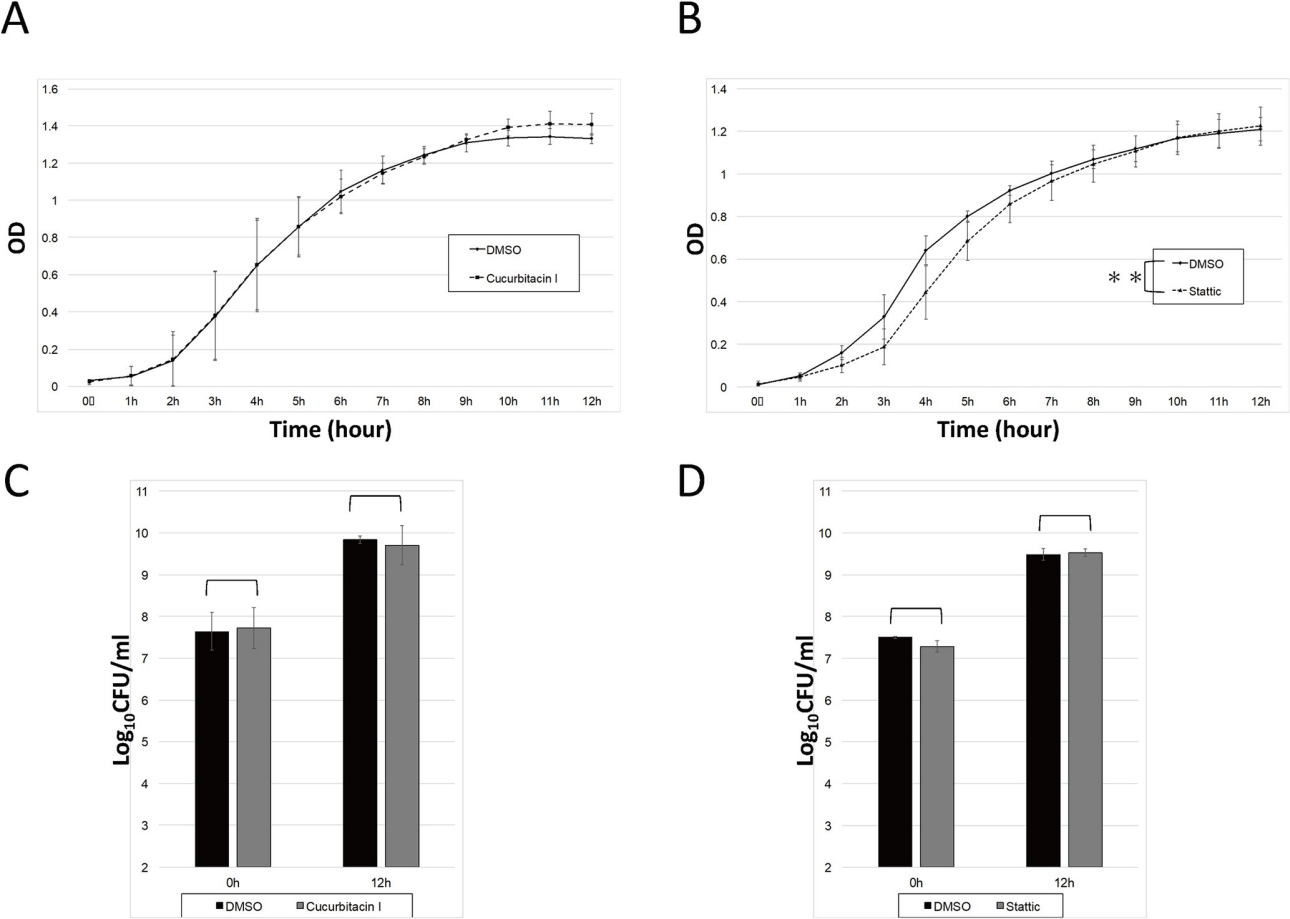
Growth of *F*. *novicida* in culture medium with inhibitors. *F*. *novicida* was cultured in BHIc with cucurbitacin I (A, C) or Stattic (B, D) and optical density (λ = 595 nm) was measured at the indicated time point (A, B). The number of CFU at 0 and 12 h was counted (C, D). Data represent the average and standard deviation of three identical experiments. Differences were analyzed with multiple comparison (Tukey–Kramer method) (A, C) or Student t-test (B, D) and indicated by asterisks, *P < 0.05.

**Fig 3:**
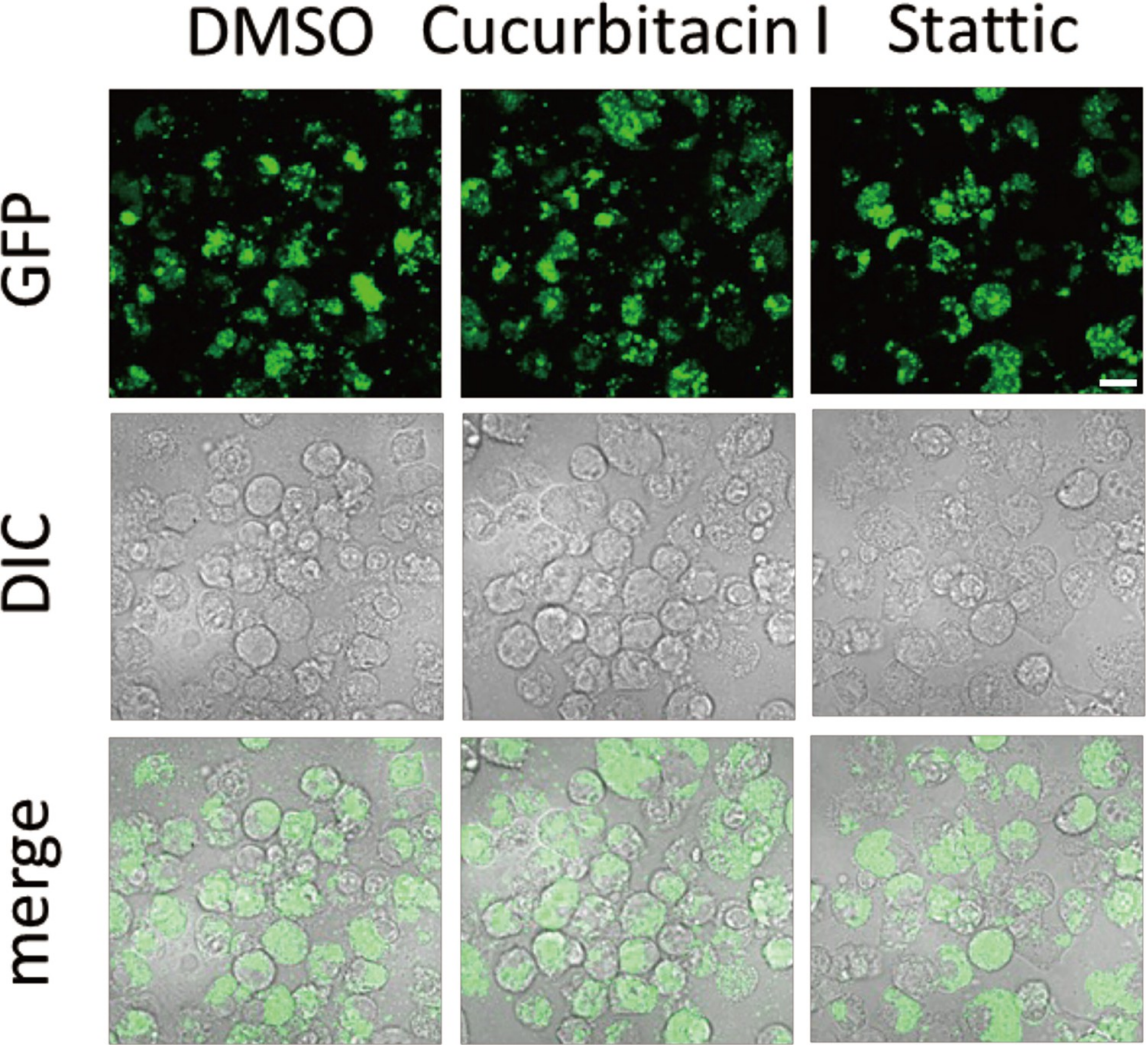
Intracellular growth of *F*. *novicida* cultured with inhibitors. *F*. *novicida* was cultured in BHIc with cucurbitacin I or Stattic. J774.1 cells were infected with inhibitor-treated *F*. *novicida* and observed at 12 h post infection. Scale bar: 10μm.

To check the direct effect of inhibitors on the growth of *F*. *novicida*, cucurbitacin I or Stattic was added into the growth medium, and the growth was measured by optical density and colony count. No significant differences in counts were observed between inhibitors and DMSO control (Fig 2A–2D). GFP-expressing *F*. *novicida* cultured with inhibitors were washed and infected into J774.1 cells, and intracellular bacteria were observed using confocal microscopy. The same levels of intracellular *F*. *novicida* was observed in bacteria cultured with cucurbitacin I or Stattic compared with that of DMSO control (Fig 3). Therefore, cucurbitacin I and Stattic had no direct effect on the growth and infectivity of *F*. *novicida*.

### Cell adhesion, invasion, and intracellular proliferation of *F*. *novicida*

**Fig 4:**
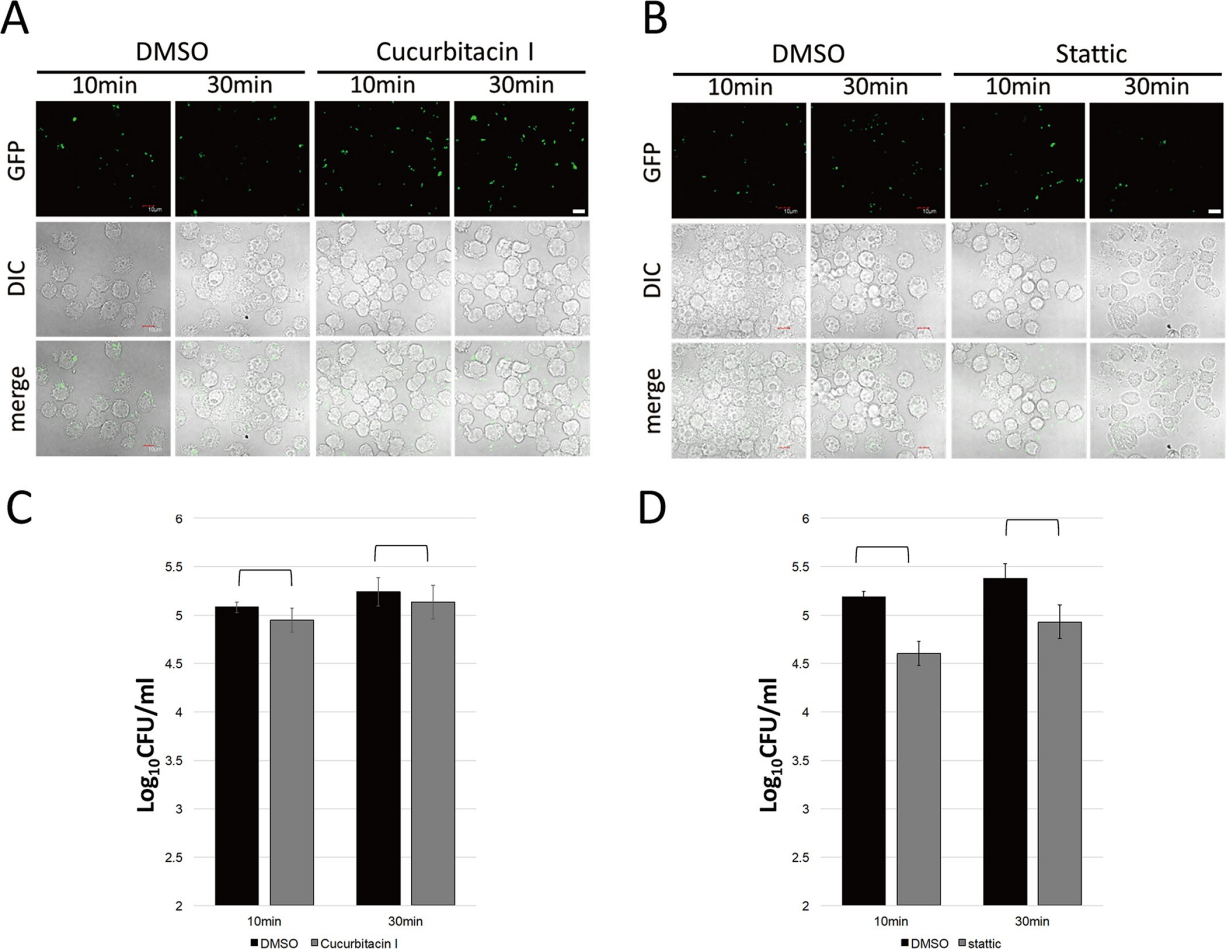
Attachment of *F*. *novicida* to cell surface. J774.1 cells treated with 1 μM of cucurbitacin I (A, C) or 10 μM Stattic (B, D) were infected with *F*. *novicida* (C, D) or GFP-expressing *F*. *novicida* (A, B). Cells were observed with confocal microscopy (A, B), or the intracellular bacterial number was counted (C, D) at 10 or 30 min post infection. The data represent the averages and standard deviations of three identical experiments. Differences were analyzed with Student t-test and indicated by asterisks, *P < 0.05. Scale bar: 10μm.

**Fig 5:**
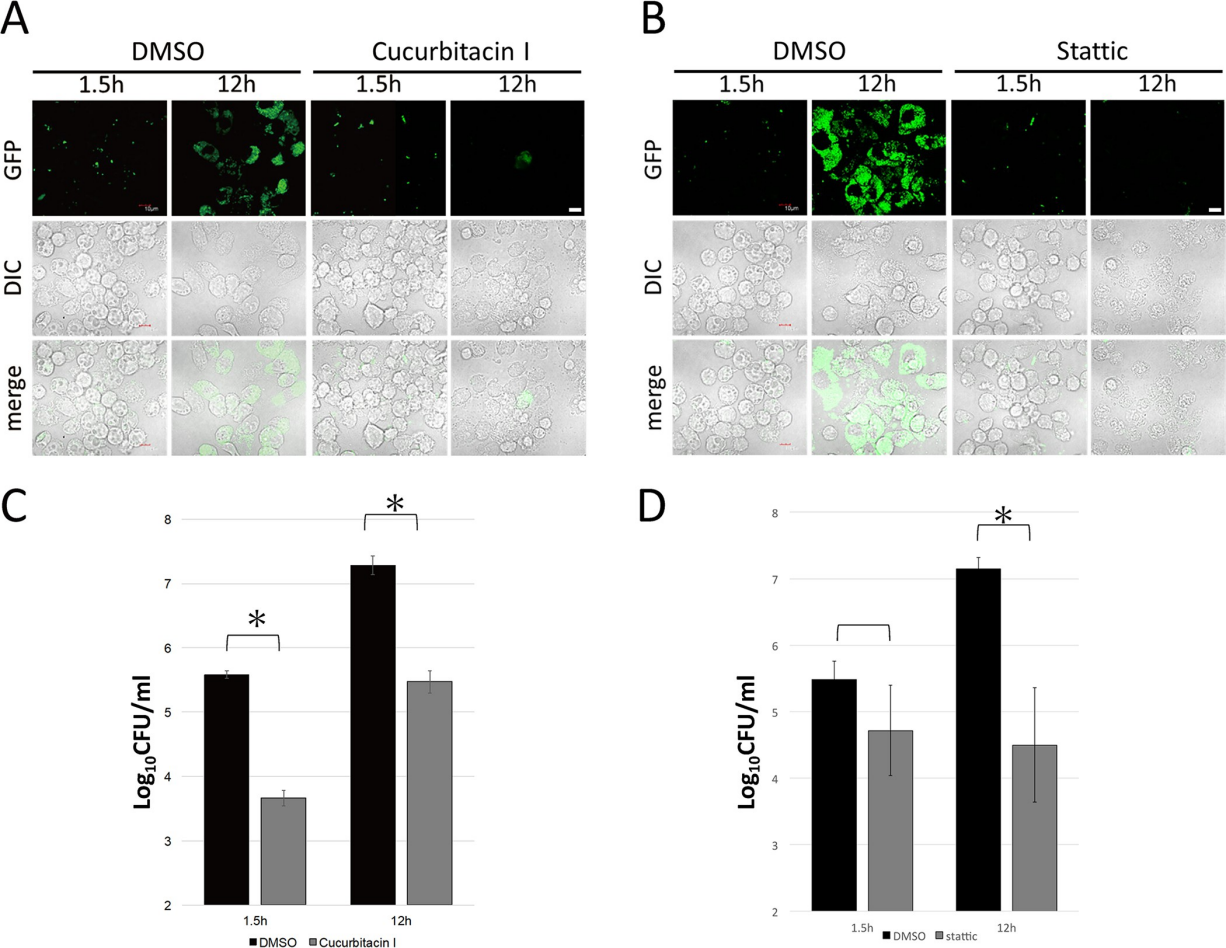
Internalization and proliferation of *F*. *novicida*. J774.1 cells treated with 1 μM of cucurbitacin I (A, C) or 10 μM of Stattic (B, D) were infected with *F*. *novicida* (C, D) or GFP-expressing *F*. *novicida* (A, B). Cells were treated with gentamicin and incubated for 1.5 or 12 h, Cells were then observed with confocal microscopy (A, B), or the intracellular bacterial number was counted (C, D). Data represent the average and standard deviation of three identical experiments. Differences were analyzed with Student t-test and indicated by asterisks, **P < 0.01, *P < 0.05. Scale bar: 10μm.

**Fig 6:**
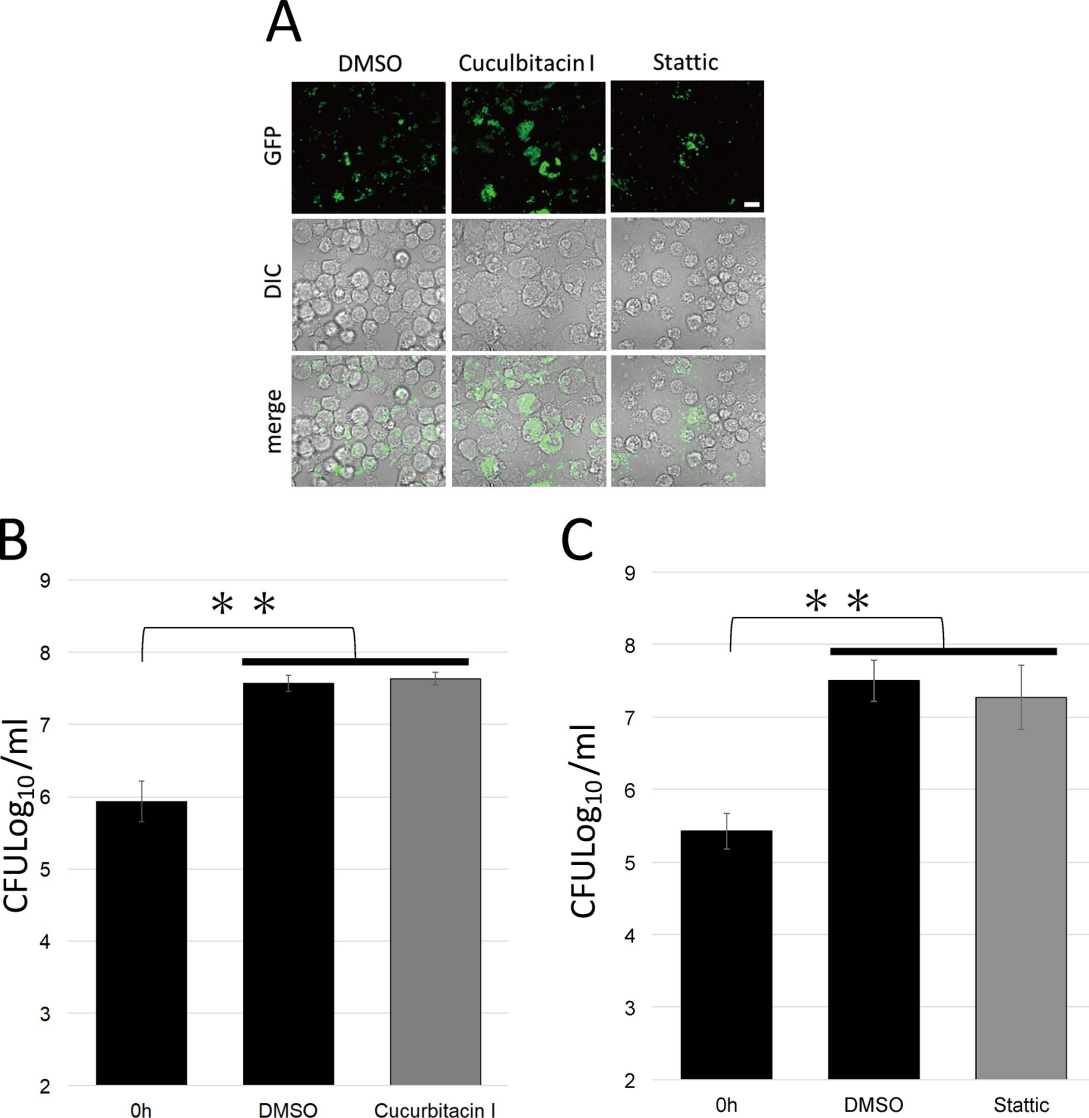
Intracellular proliferation of *F*. *novicida*. J774.1 cells were infected with *F*. *novicida* (B, C) or GFP-expressing *F*. *novicida* (A). Cells were treated with gentamicin and cultured with 1 μM of cucurbitacin I (A, B) or 10 μM of Stattic (A, C) for 12 h. Cells were observed with confocal microscopy(A), or the intracellular bacterial number was counted (B, C). Data represent the average and standard deviation of three identical experiments. Differences were analyzed with multiple comparison (Dunnett’s test) and indicated by asterisks, **P < 0.01. Scale bar: 10μm.

Infection of *F*. *novicida* is established via three infections steps: attachment, internalization, and proliferation. To investigate which step of infection is affected by the identified inhibitors, the attachment of *F*. *novicida* was initially tested. J774.1 cells were treated with cucurbitacin or Stattic and then infected with *F*. *novicida*. The number of bacteria attached to cells just after infection (10 and 30 min) was measured by colony counting. No significant difference was observed in colony counts between control DMSO and inhibitor treatments (Fig 4). Next, to test the effect of inhibitors of internalization and proliferation, J774.1 cells were treated with inhibitors and infected with *F*. *novicida*. Cells were incubated for 1 h to allow internalization of bacteria while attached bacterial cells were removed by gentamicin treatment. After 1.5 and 12 h incubation, the number of internalized and proliferated *F*. *novicida* were measured. The number of bacteria was decreased in cells treated with each inhibitor compared with that treated with DMSO, indicating that either internalization or proliferation were affected by the inhibitors (Fig 5). To determine which steps of internalization and proliferation was affected by inhibitors, the proliferation of *F*. *novicida* was examined. J774.1 cells were infected with *F*. *novicida* and incubated for 1 h to allow internalization of bacteria. Attached bacteria were removed by gentamicin treatment and infected cells were treated with inhibitors. The number of bacteria in inhibitor-treated cells were unaffected by inhibitors (Fig 6). Thus, cucurbitacin I and Stattic affect internalization but not proliferation in *F*. *novicida* infection.

### Phagocytotic activity

**Fig 7:**
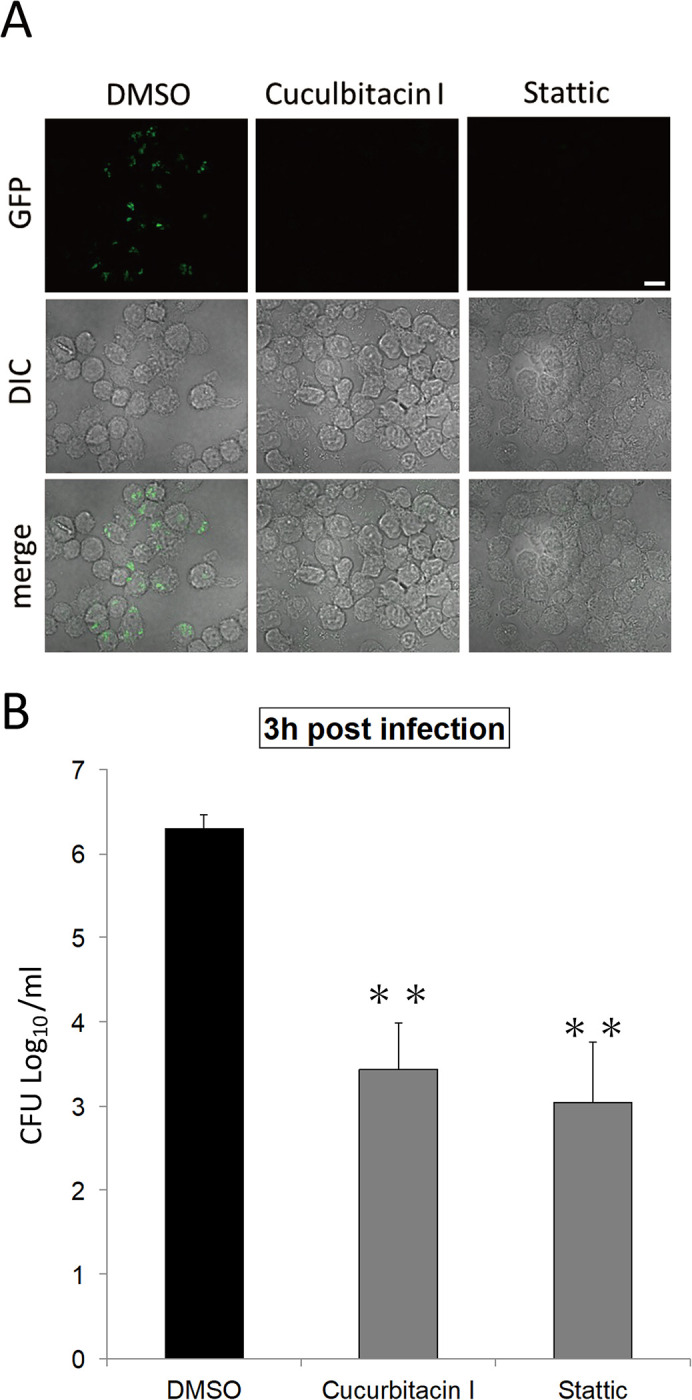
Effects of inhibitors on phagocytosis. J774.1 cells treated with 1 μM of cucurbitacin I (A, B) or 10 μM of Stattic (A, B) were infected with *E*. *coli* (B) or GFP-expressing *E*.*coli* (A). Cells were treated with gentamicin and incubated for 3 h. Cells were observed with confocal microscopy (A), or the intracellular bacterial number was counted (B). Data represent the average and standard deviation of three identical experiments. Differences were analyzed with multiple comparison (Dunnett’s test) and indicated by asterisks, **P < 0.01. Scale bar: 10μm.

As cucurbitacin I and Stattic affected the internalization of *F*. *novicida*, the effect of inhibitors on phagocytosis was investigated using the nonintracellular bacteria *E*. *coli*. J774.1 cells were treated with cucurbitacin I or Stattic and infected with *E*. *coli*. Cells were incubated for 3 h to allow phagocytosis and attached cells were removed by gentamicin treatment for 30 min. At 3.5 h post infection, the internal bacterial number was measured by colony counting. Addition of inhibitors decreased the number of internalized *E*. *coli* (Fig 7), indicating cucurbitacin I and Stattic affect host cell phagocytosis.

### Actin filaments

**Fig 8:**
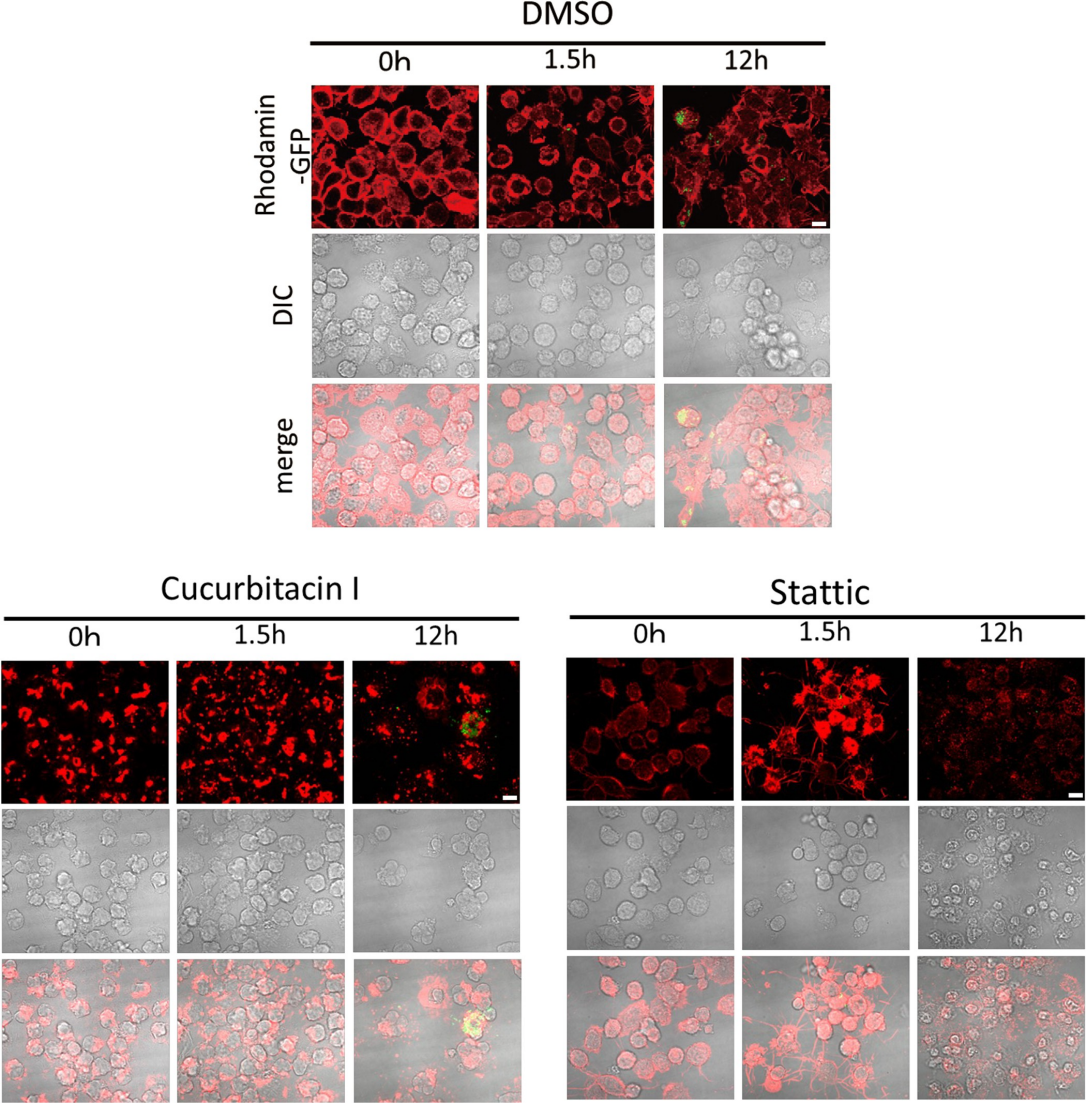
Effects of inhibitors on actin filaments. J774.1 cells treated with 1 μM of cucurbitacin I or 10 μM of Stattic were infected with GFP-expressing *F*. *novicida* for the indicated time. Cells were stained with phalloidin-rhodamine and observed by confocal microscopy. Scale bar: 10μm.

Phagocytosis results from polymerization, depolymerization, and rearrangement of actin [23]. To investigate the effect of inhibitors on actin polymerization, J774.1 cells were treated with cucurbitacin I or Stattic for 2 h and infected with *F*. *novicida*, and then actin was visualized using immunofluorescence microscopy. Abnormal arrangements of actin were observed in inhibitor-treated cells compared with those in cells treated with DMSO control (Fig 8). These results suggest that the correct arrangement of actin is crucial for the infection of *F*. *novicida*.

## Discussion

To identify the host factors important for *Francisella* infection, 368 inhibitors were screened, and those that affected *F*. *novicida* infection were selected. Consequently, 56 compounds inhibited the infection of *F*. *novicida* while eight enhanced the infection. In this study we focused on 56 inhibitors that negatively affected infection to identify the host factors important for infection. Most of the 56 inhibitors possessed antibiotic property, whereas three inhibitors were related to the JAK2/STAT3 pathway. Therefore, we focused on the inhibitors related to the JAK2/STAT3 pathway without antibiotic properties, and cucurbitacin I was selected for farther study.

Cucurbitacin I is a triterpenoid compound derived from the fruit extract of plants, such as cucumber, in the *Cucurbitaceae* family [24]. Cucurbitacin I inhibits JAK2 phosphorylation and thereby suppresses the levels of tyrosine-phosphorylated STAT3 [17]. In this study, cucurbitacin I inhibited the infection of *F*. *novicida* but failed to affect the growth of *F*. *novicida* in culture medium. To confirm the involvement of the JAK2/STAT3 pathway in *F*. *novicida* infection, another inhibitor of the JAK2/STAT3 pathway, Stattic was tested. Stattic is a nonpeptide small molecule that inhibits the dimerization of STAT3 through the SH2 domain [25]. Similar to cucurbitacin I, Stattic did not affect growth in the culture medium but did inhibit *F*. *novicida* infection. In addition, *F*. *novicida* infection tended to enhance activation of STAT3 just after infection. These results indicate that the JAK2/STAT3 pathway plays an important role in *F*. *novicida* infection.

To examine which of the three steps of adhesion, invasion, and intracellular proliferation is inhibited by cucurbitacin I or Stattic, cells were treated with inhibitors and the subsequent effects observed at different time points. Treatment of inhibitors failed to affect the attachment of *F*. *novicida* to the cells at 10 or 30 min post infection, indicating that cucurbitacin I and Stattic affects the internalization or intracellular proliferation. Cucurbitacin I or Stattic treatment after infection failed to decrease the number of intracellular *F*. *novicida*, indicating that intracellular proliferation was not affected by the inhibitors, indicating that the JAK2/STAT3 pathway is important for the internalization step of *F*. *novicida*. These results are consistent with the various reports concerning intracellular infection by other bacteria. In infection by *Brucella abortus*, the AK2/STAT3 pathway is important for the intracellular survival of the bacteria [26]. In *Helicobacter pylori* infection, inhibition of the JAK2/STAT3 pathway reduces the development of gastric cancer [27]. In addition, the JAK2/STAT3 pathway is important for the development of pulmonary fibrosis in *Mycobacterium tuberculosis* infection [28]. Thus, the JAK2/STAT3 pathway is important for the infection and pathogenesis of various bacterial intracellular infections.

*Francisella* are ingested through the pseudopodia of macrophages and incorporated into spacious vacuoles with endosomal markers [7, 8]. The organism then escapes from the phagosomal membrane and replicates in the cytoplasm [9]. To examine which step of phagocytosis or escape from phagosome is the target of inhibitors, the ingestion of *E*. *coli*, a bacterium that cannot escape from the phagosome was evaluated. Subsequently, the number of ingested intracellular *E*. *coli* was also decreased by treatment with cucurbitacin I or Stattic. This result indicates that cucurbitacin I and Stattic inhibit the phagocytosis step of bacterial infection.

Phagocytosis results from polymerization, depolymerization, and rearrangement of actin [23], and we therefore evaluated the actin dynamics of *F*. *novicida*-infected cells and observed abnormal arrangements of actin in cucurbitacin I- or Stattic-treated cells. These results suggest that the JAK2/STAT3 pathway regulates actin dynamics followed by phagocytosis. This finding is consistent with a previous study where cucurbitacin I inhibited cell motility or proliferation of cancer cells by interfering with actin dynamics [29, 30].

Since cucurbitacin I exhibits an antitumor effect, cucurbitacin I and JAK2/STAT3 inhibitors have received increasing attention as potential cancer therapeutic agents [31, 32]. In this study, we identified cucurbitacin I as an inhibitor of *F*. *novicida* infection and demonstrated that the JAK2/STAT3 pathway is important for the actin dynamics that underlie phagocytosis. In infection by other intracellular bacteria such as *Brucella* and *Mycobacterium*, the JAK2/STAT3 pathway is important for the intracellular growth and pathogenesis [26, 28]. Moreover, cucurbitacin I exhibits an antimicrobial effect through induction of autophagy [33]. Therefore, inhibitors such as cucurbitacin I and Stattic can be utilized as antimicrobial agents, and the JAK2/STAT3 pathway can be a therapeutic target of infection with intracellular bacteria as well.

## Supporting information

S1 Table List of inhibitors. *Fluorescence intensity of GFP-expressing *F*. *novicida* >5000 higher than that of the DMSO control was determined as positively regulating inhibitors (+), and >4000 lower than that of the control was determined as negatively regulating inhibitors (−). **Same compounds but derived from different providers.
(PDF)

S1 Data (XLSX)

## References

[1] Ellis J, Oyston PC, Green M, Titball RW. Tularemia. Clin Microbiol Rev. 2002;15(4):631–46. Epub 2002/10/05. doi: 10.1128/CMR.15.4.631-646.2002 ; PubMed Central PMCID: PMC126859. 12364373 PMC126859

[2] McLendon MK, Apicella MA, Allen LA. *Francisella tularensis*: taxonomy, genetics, and Immunopathogenesis of a potential agent of biowarfare. Annu Rev Microbiol. 2006;60:167–85. Epub 2006/05/18. doi: 10.1146/annurev.micro.60.080805.142126 ; PubMed Central PMCID: PMC1945232. 16704343 PMC1945232

[3] Maurin M. *Francisella tularensis* as a potential agent of bioterrorism? Expert Rev Anti Infect Ther. 2015;13(2):141–4. Epub 2014/11/22. doi: 10.1586/14787210.2015.986463 . 25413334

[4] Anthony LD, Burke RD, Nano FE. Growth of Francisella spp. in rodent macrophages. Infect Immun. 1991;59(9):3291–6. Epub 1991/09/01. doi: 10.1128/iai.59.9.3291-3296.1991 ; PubMed Central PMCID: PMC258167. 1879943 PMC258167

[5] Kingry LC, Petersen JM. Comparative review of *Francisella tularensis* and *Francisella novicida*. Front Cell Infect Microbiol. 2014;4:35. Epub 2014/03/25. doi: 10.3389/fcimb.2014.00035 ; PubMed Central PMCID: PMC3952080. 24660164 PMC3952080

[6] Broms JE, Sjostedt A, Lavander M. The Role of the *Francisella Tularensis* Pathogenicity Island in Type VI Secretion, Intracellular Survival, and Modulation of Host Cell Signaling. Front Microbiol. 2010;1:136. Epub 2010/01/01. doi: 10.3389/fmicb.2010.00136 ; PubMed Central PMCID: PMC3109350. 21687753 PMC3109350

[7] Clemens DL, Lee BY, Horwitz MA. Virulent and avirulent strains of *Francisella tularensis* prevent acidification and maturation of their phagosomes and escape into the cytoplasm in human macrophages. Infect Immun. 2004;72(6):3204–17. Epub 2004/05/25. doi: 10.1128/IAI.72.6.3204–3217.2004 ; PubMed Central PMCID: PMC415696. 15155622 PMC415696

[8] Clemens DL, Lee BY, Horwitz MA. *Francisella tularensis* enters macrophages via a novel process involving pseudopod loops. Infect Immun. 2005;73(9):5892–902. Epub 2005/08/23. doi: 10.1128/IAI.73.9.5892–5902.2005 ; PubMed Central PMCID: PMC1231130. 16113308 PMC1231130

[9] Golovliov I, Baranov V, Krocova Z, Kovarova H, Sjostedt A. An attenuated strain of the facultative intracellular bacterium *Francisella tularensis* can escape the phagosome of monocytic cells. Infect Immun. 2003;71(10):5940–50. Epub 2003/09/23. doi: 10.1128/IAI.71.10.5940–5950.2003 ; PubMed Central PMCID: PMC201066. 14500514 PMC201066

[10] Checroun C, Wehrly TD, Fischer ER, Hayes SF, Celli J. Autophagy-mediated reentry of *Francisella tularensis* into the endocytic compartment after cytoplasmic replication. Proc Natl Acad Sci U S A. 2006;103(39):14578–83. Epub 2006/09/20. doi: 10.1073/pnas.0601838103 ; PubMed Central PMCID: PMC1600002. 16983090 PMC1600002

[11] Chong A, Celli J. The francisella intracellular life cycle: toward molecular mechanisms of intracellular survival and proliferation. Front Microbiol. 2010;1:138. Epub 2010/01/01. doi: 10.3389/fmicb.2010.00138 ; PubMed Central PMCID: PMC3109316. 21687806 PMC3109316

[12] Chong A, Wehrly TD, Child R, Hansen B, Hwang S, Virgin HW, et al. Cytosolic clearance of replication-deficient mutants reveals *Francisella tularensis* interactions with the autophagic pathway. Autophagy. 2012;8(9):1342–56. Epub 2012/08/07. doi: 10.4161/auto.20808 ; PubMed Central PMCID: PMC3442881. 22863802 PMC3442881

[13] Hofmann HD, Kirsch M. JAK2-STAT3 signaling: A novel function and a novel mechanism. JAKSTAT. 2012;1(3):191–3. Epub 2012/07/01. doi: 10.4161/jkst.20446 ; PubMed Central PMCID: PMC3670243. 24058769 PMC3670243

[14] Wu H, Huang M, Cao P, Wang T, Shu Y, Liu P. MiR-135a targets JAK2 and inhibits gastric cancer cell proliferation. Cancer Biol Ther. 2012;13(5):281–8. Epub 2012/02/09. doi: 10.4161/cbt.18943 . 22310976

[15] Kiu H, Nicholson SE. Biology and significance of the JAK/STAT signalling pathways. Growth Factors. 2012;30(2):88–106. Epub 2012/02/22. doi: 10.3109/08977194.2012.660936 ; PubMed Central PMCID: PMC3762697. 22339650 PMC3762697

[16] Murray PJ. The JAK-STAT signaling pathway: input and output integration. J Immunol. 2007;178(5):2623–9. Epub 2007/02/22. doi: 10.4049/jimmunol.178.5.2623 . 17312100

[17] Blaskovich MA, Sun J, Cantor A, Turkson J, Jove R, Sebti SM. Discovery of JSI-124 (cucurbitacin I), a selective Janus kinase/signal transducer and activator of transcription 3 signaling pathway inhibitor with potent antitumor activity against human and murine cancer cells in mice. Cancer Res. 2003;63(6):1270–9. Epub 2003/03/22. . 12649187

[18] Mc Gann P, Rozak DA, Nikolich MP, Bowden RA, Lindler LE, Wolcott MJ, et al. A novel brain heart infusion broth supports the study of common *Francisella tularensis* serotypes. J Microbiol Methods. 2010;80(2):164–71. Epub 2009/12/17. doi: 10.1016/j.mimet.2009.12.005 . 20005265

[19] Nagle SC Jr., Anderson RE, Gary ND. Chemically defined medium for the growth of *Pasteurella tularensis*. J Bacteriol. 1960;79(4):566–71. Epub 1960/04/01. doi: 10.1128/jb.79.4.566–571.1960 ; PubMed Central PMCID: PMC278733. 14425793 PMC278733

[20] Nakamura T, Shimizu T, Inagaki F, Okazaki S, Saha SS, Uda A, et al. Identification of Membrane-Bound Lytic Murein Transglycosylase A (MltA) as a Growth Factor for *Francisella novicida* in a Silkworm Infection Model. Front Cell Infect Microbiol. 2020;10:581864. Epub 2021/02/09. doi: 10.3389/fcimb.2020.581864 ; PubMed Central PMCID: PMC7862118. 33553001 PMC7862118

[21] Chen K, Yoshimura T, Gong W, Tian C, Huang J, Trinchieri G, et al. Requirement of CRAMP for mouse macrophages to eliminate phagocytosed *E*. *coli* through an autophagy pathway. J Cell Sci. 2021;134(5). Epub 2021/01/21. doi: 10.1242/jcs.252148 ; PubMed Central PMCID: PMC7970306. 33468624 PMC7970306

[22] Drevets DA, Canono BP, Campbell PA. Measurement of bacterial ingestion and killing by macrophages. Curr Protoc Immunol. 2015;109:14 6 1–6 7. Epub 2015/04/08. doi: 10.1002/0471142735.im1406s109 . 25845563

[23] May RC, Machesky LM. Phagocytosis and the actin cytoskeleton. J Cell Sci. 2001;114(Pt 6):1061–77. Epub 2001/03/03. doi: 10.1242/jcs.114.6.1061 . 11228151

[24] Cai Y, Fang X, He C, Li P, Xiao F, Wang Y, et al. Cucurbitacins: A Systematic Review of the Phytochemistry and Anticancer Activity. Am J Chin Med. 2015;43(7):1331–50. Epub 2015/10/28. doi: 10.1142/S0192415X15500755 . 26503558

[25] Schust J, Sperl B, Hollis A, Mayer TU, Berg T. Stattic: a small-molecule inhibitor of STAT3 activation and dimerization. Chem Biol. 2006;13(11):1235–42. Epub 2006/11/23. doi: 10.1016/j.chembiol.2006.09.018 . 17114005

[26] Yi J, Wang Y, Zhang J, Xu J, Li T, Chen C. Effects of JAK2 / STAT3 Signaling Pathway Activation on Intracellular Survival of *Brucella*. Pak Vet J. 2018;38(2):153–8. doi: 10.29261/pakvetj/2018.048

[27] Judd LM, Menheniott TR, Ling H, Jackson CB, Howlett M, Kalantzis A, et al. Inhibition of the JAK2/STAT3 pathway reduces gastric cancer growth in vitro and in vivo. PLoS One. 2014;9(5):e95993. Epub 2014/05/09. doi: 10.1371/journal.pone.0095993 ; PubMed Central PMCID: PMC4013079. 24804649 PMC4013079

[28] Izuhara K, Conway SJ, Moore BB, Matsumoto H, Holweg CT, Matthews JG, et al. Roles of Periostin in Respiratory Disorders. Am J Respir Crit Care Med. 2016;193(9):949–56. Epub 2016/01/13. doi: 10.1164/rccm.201510-2032PP ; PubMed Central PMCID: PMC4872656. 26756066 PMC4872656

[29] Guo H, Kuang S, Song QL, Liu M, Sun XX, Yu Q. Cucurbitacin I inhibits STAT3, but enhances STAT1 signaling in human cancer cells in vitro through disrupting actin filaments. Acta Pharmacol Sin. 2018;39(3):425–37. Epub 2017/11/10. doi: 10.1038/aps.2017.99 ; PubMed Central PMCID: PMC5843842. 29119966 PMC5843842

[30] Knecht DA, LaFleur RA, Kahsai AW, Argueta CE, Beshir AB, Fenteany G. Cucurbitacin I inhibits cell motility by indirectly interfering with actin dynamics. PLoS One. 2010;5(11):e14039. Epub 2010/12/03. doi: 10.1371/journal.pone.0014039 ; PubMed Central PMCID: PMC2991314. 21124831 PMC2991314

[31] Lee DH, Iwanski GB, Thoennissen NH. Cucurbitacin: ancient compound shedding new light on cancer treatment. ScientificWorldJournal. 2010;10:413–8. Epub 2010/03/09. doi: 10.1100/tsw.2010.44 ; PubMed Central PMCID: PMC5763727. 20209387 PMC5763727

[32] Alghasham AA. Cucurbitacins—a promising target for cancer therapy. Int J Health Sci (Qassim). 2013;7(1):77–89. Epub 2013/04/06. doi: 10.12816/0006025 ; PubMed Central PMCID: PMC3612419. 23559908 PMC3612419

[33] Wu Y, Chen H, Li R, Wang X, Li H, Xin J, et al. Cucurbitacin-I induces hypertrophy in H9c2 cardiomyoblasts through activation of autophagy via MEK/ERK1/2 signaling pathway. Toxicol Lett. 2016;264:87–98. Epub 2016/11/12. doi: 10.1016/j.toxlet.2016.11.003 . 27836799

