# Supplementary material for: Role of the JAK2/STAT3 pathway on infection of Francisella novicida
Source: PLoS One. 2024 Sep 10;19(9):e0310120. doi: 10.1371/journal.pone.0310120 (PMC11386456; doi:10.1371/journal.pone.0310120)
Supplement: S1 Table — *Fluorescence intensity of GFP-expressing F. novicida >5000 higher than that of the DMSO control was determined as positively regulating inhibitors (+), and >4000 lower than that of the control was determined as negatively regulating inhibitors (−). **Same compounds but derived from different providers. (PDF) [file pone.0310120.s001.pdf]

S1 Table. List of inhibitors.

| Compound | Category | Effect on infection* |
|---|---|---|
| (+)-JQ1 | BRD4 bromodomain | |
| 1-Azakenpaullone | GSK | |
| 1400W, HCl | iNOS | |
| 17-AAG | HSP90 | |
| 2',5'-dideoxyadenosine | adenylcyclase | |
| 3-ATA | CDK4 | + |
| 4-cyano-3-methylisoquinoline | PKA | |
| 5-FU | antitumor (thymidylate synthetase) | |
| 5,15-DPP | STAT3 | - |
| a-Amanitin | RNA polymerase | |
| A23187 | Ca ionophore | |
| A83-01 | ALK | |
| ABT-702 | AK | - |
| ABT-737 | blc-2 | |
| ABT-888 (Veliparib) | PARP | |
| Aclarubicin | antitumor (topo I/II) | |
| Actinomycin D | antitumor (RNA) | |
| Actinonin | aminopeptidase M | - |
| AG014699 (Rucaparib) | PARP | |
| AG1024** | IGF-1R | |
| AG1024** | IGF-IR | |
| AG1296** | PDGFR | |
| AG1296** | PDGFR | |
| AG1478** | EGFR | |
| AG1478** | EGFR | |
| AG490** | Jak-2 | |
| AG490** | EGFR | |
| AG825** | HER2 (erbB2/neu), EGFR | |
| AG825** | HER2 | |
| AG957** | Bcr-abl | - |
| AG957** | Bcr-Abl | |
| AGL 2263 | IGF-IR | |
| AKT inhibitor | AKT | |
| Akt Inhibitor IV | AKT | |
| Akt Inhibitor VIII, Isozyme-Selective, Akti-1/2 | AKT | |
| Akt Inhibitor XI | AKT | |
| ALLN | calpain, cathepsin B, L | |
| Alsterpaullone, 2-cyanoethyl | CDK | |
| Amastatin | aminopeptidase A | - |
| AMD3100 octahydrochloride | CXCR4 | - |
| AMI-1 | PRMT1 | |
| Amiloride | Na channel | |
| Aminoglutethimide | aromatase | - |
| Aminoguanidine, HCl | NOS | |
| AMT, HCl | iNOS | + |
| Anacardic acid | HAT | |
| anisomycin | stress inducer | - |
| Antimycin A1 | mitochondrial complex III | |
| Aphidicolin | DNA polymerase | |
| ATM kinase inhibitor | ATM | |
| ATM/ATR kinase inhibitor | ATM | |
| Aurora kinase inhibitor II | Aurora | |
| Aurora kinase inhibitor III | Aurora | |
| Aurora kinase/cdk inhibitor | Aurora | |
| Axitinib | Multi-kinases | |
| AY 9944 | Hedgehog | |
| Azacytidine | DNA methyltransferase | |
| AZT | reverse transcriptase | |
| b-Rubromycin | telomerase | |
| BADGE | PPAR-g | |
| Bafilomycin A1 | V-ATPase | - |
| Baicalein | 12-lipoxygenase | |
| Benzamide | PARP-1 | |
| Benzylguanine | O6-methylguanine-DNA methyltransferase (MGMT) | |
| Bestatin | antitumor (aminopeptidase B) | + |
| BH3I-1 | Bcl-XL | - |
| BIO | GSK-3 | |

| | | |
|---|---|---|
| Bisindolymaleimide I, HCl** | PKC | |
| Bisindolymaleimide I, HCl** | PKC | |
| BIX01294 | G9a | |
| Bleomycin sulfate | antitumor (DNA) | |
| BMS-345541 | IKK | |
| bortezomib | Proteasome | - |
| BPIQ-II | EGFR | |
| brefeldin A | golgi inhibitor | - |
| BSI-201 (Iniparib) | PARP | |
| C646 | p300 | |
| C75 | fatty acid synthase (FAS) | |
| CA-074 | cathepsin B | - |
| Camptothecin | antitumor (topo I) | |
| Cantharidin | PP2A | |
| CCG-1423 | Rho/SRF | |
| Cdk1/2 inhibitor III | CDK | |
| Cdk2/9 inhibitor | CDK | |
| Cdk4 inhibitor | CDK | - |
| Cerulenin | FAS | |
| cFMS Receptor Tyrosine Kinase Inhibitor | Fms | |
| Chetomin | HIF | - |
| Chk2 inhibitor | Chk | |
| Chk2 inhibitor II | Chk | |
| chlorpromazine hydrochloride | antipsychotic drug | |
| Cisplatin | antitumor (DNA) | |
| Clofibrate | PPAR-a activator | |
| compound C | AMPK | |
| cPLA2inhibitor | PLA2 | |
| crizotinib | EML4-ALK | |
| CT99021 | GSK-3 | |
| Cucurbitacin I | Jak-2 | - |
| Cycloheximide | protein synthesis | |
| cyclopamine | Hedgehog | - |
| Cyclosporin A | PP2B/cyclophilin | |
| Cytochalasin D | actin filament | - |
| Cytostatin | PP2A | |
| D4476 | CK | |
| D609 | PC-PLC | |
| Damnacanthal** | lck (p56), TYK | |
| Damnacanthal** | Lck | |
| DAPT | Notch | |
| dasatinib | Bcr-Abl/Src | |
| Daunorubicin, HCl | antitumor (DNA) | |
| Debromohymenialdisine (DBH) | Chk 1, 2 | |
| Decitabine | DNMT | |
| Decylubiquinone | mitochondrial permeability transition pore (MPTP) | |
| Deoxynojirimycin | glucosidase I, II | |
| Dephostatin | tyr phosphatase (PTP) | |
| Deprenyl | monoamine oxdase B | |
| Dequalinium | K channel | |
| desipramine hydrochloride | depression treatment | |
| Dexamethasone | GR | + |
| DFMO | ornithine decarboxylase (ODC) | |
| Diacylglycerol kinase inhibitor II | DGK | |
| Diazoxide | K channel opener | |
| DIDS | Cl channel | |
| Diltiazem | Ca channel | |
| Dimethyloxalylglycine | HIF-1a hydroxylase | |
| Dioctanoylglycol | DAG kinase | |
| DMAT | CK | |
| Doxorubicin, HCl | antitumor (DNA) | |
| E-64d | calpain | - |
| Ellagic acid | CK | |
| ENMD-2076 | Aurora | |
| ERK inhibitor II | MAPK | |
| erlotinib | EGFR | |
| Etoposide (VP-16) | antitumor (topo II) | |
| ETYA | 12, 15-lipoxygenase | |
| everolimus | mTOR | |
| FH535 | Wnt | |
| Finasteride | 5α-reductase | |

| | | |
|---|---|---|
| FK-506 | PP2B/FKBP | |
| Flt-3 Inhibitor | Flt-3 | |
| Flutamide | antitumor (AR) | |
| Formestane | aromatase | - |
| FTI-276 | farnesyltransferase | |
| Fumagillin | methionine aminopeptidase | |
| Fumitremorgin C | BCRP | |
| Fumonisin B1 | sphingosine N-acyltransferase | |
| Gant61 | GLI1 | |
| gefitinib | EGFR | - |
| Genistein | EGFR, topoII | |
| GGTI-286 | geranylgeranyltransferase I | |
| Glibenclamide | K channel | - |
| GM 6001 | MMP | |
| Go7874 | PKC | |
| GSK-3 inhibitor II | GSK-3 | |
| GSK-3 inhibitor IX | GSK | |
| H-1152 | ROCK | |
| H-7 | PKC, PKA | |
| H-89 | PKA | |
| H-89, HCl | PKA | |
| HA 14-1 | Bcl-2 | - |
| HA1077 | ROCK | |
| HR22C16 | kinesin Eg5 | |
| Hydroxyurea | ribonucleotide reductase | |
| IBMX | PDE | |
| IC60211 | DNA-PK | |
| IKK-2 inhibitor VI | IKK | |
| imatinib mesylate | Bcr-Abl/Kit | - |
| indirubin-3'-monoxime | GSK | |
| Ionomycin | Ca ionophore | |
| IRAK-1/4 inhibitor | IRAK | |
| isogranulatimide | Chk | |
| IWP-2 | Wnt | |
| IWR-1-endo | Wnt | |
| JAK Inhibitor I | Jak | |
| JAK3 Inhibitor VI | Jak | |
| Jervine | Hedgehog | - |
| JNK inhibitor VIII | JNK | |
| Kenpaullone | CDC2 | |
| Kenpaullone | CDK | |
| KN-62 | CAMKII | |
| KN-93** | CAMKII | |
| KN93** | CAMKII | + |
| KT 5823** | PKG | |
| KT5823** | PKG | - |
| L-NMMA | NOS | |
| Lactacystin | proteasome | |
| lapatinib | EGFR/Her2 | |
| Lavendustin C | CAMKII | |
| LDN193189 | ALK | |
| lenalidomide | thalidomide family | |
| Leptomycin B | CRM1 | - |
| LFM-A13** | Burton's tyrosine kinase(BTK) | - |
| LFM-A13** | BTK | |
| Lidocaine | Na channel | |
| Lonidamine | MPTP opener | |
| Lovastatin | HMG-CoA reductase | |
| LY 83583 | guanylate cyclase | |
| LY-294002 | PI3K | |
| LY2157299 | TGFb-R | |
| LY294002 | PI3K | |
| Manumycin A | farnesyltransferase | |
| MDM2 inhibitor | Mdm2 | |
| MDV3100 | AR | |
| MEK inhibitor I | MEK | |
| Methotrexate | antitumor (DHFR) | |
| MG-132 | proteasome | - |
| Mifepristone | progesterone receptor | - |
| Mitomycin C | antitumor (DNA) | |
| MK 886 | PPAR-a | |

| | | |
|---|---|---|
| MK-4827 (Niraparib) | PARP | |
| ML-7** | myosin light chain kinase | |
| ML-7** | MLCK | |
| MLN8237 | Aurora | |
| Monastrol | kinesin Eg5 | |
| Monensin | Na ionophore | - |
| MST-312 | telomerase | |
| N-Acetyl-L-cysteine | NF-kB | |
| N-phenylanthranilic acid | Cl channel | |
| N1,N12-Diethylspermine (BESpm) | spermidine/spermine N1-acetyltransferase (SSAT) activator | |
| Nalidixic acid | reverse transcriptase | |
| Nifedipine | Ca channel | - |
| Nigericin | K ionophore | - |
| nilotinib | Bcr-Abl | - |
| Nocodazole | tubulin depolymerization | |
| Nordihydroguaiaretic acid (NDGA) | lipoxygenase | |
| NS-398 | COX-2 | |
| NSC625987 | CDK | - |
| NSC95397 | Cdc25 | - |
| NU1025 | PARP | |
| NU6102 | CDK | |
| Nutlin-3 | Mdm2 | |
| OBAA | PLA2 | |
| ODQ | guanylate cyclase | |
| Olaparib | PARP-1/2-selective | |
| Oligomycin | F1-ATPase | |
| Olomoucine** | CDKs | |
| Olomoucine** | CDK | |
| orlistat | lipase | |
| OSI-906 | IGF-IR | |
| OSU-03012 | PDK1 | |
| Ouabain | Na/K ATPase | - |
| PAC-1 | caspase activator | |
| Paclitaxel | antitumor (tubulin) | |
| pazopanib | Multi-kinases | |
| PCI-34051 | HDAC8 | |
| PD 98059 | MEK | |
| PD169316 | p38 (MAPK) | |
| PD173074 | FGFR | |
| PD98059 | MEK | |
| PDGF receptor tyrosine kinase inhibitor IV | PDGFR | |
| PDGF receptor tyrosine kinase inhibitor V | PDGFR | |
| Pepstatin A | cathepsin D | - |
| PF-04217903 | c-Met | |
| PGP-4008 | MDR | |
| Phenelzine | monoamine oxidase | |
| Pifithrin-a (cyclic) | p53 | - |
| PIM1 Inhibitor II | PIM | - |
| PIM1/2 Kinase Inhibitor V | PIM | |
| PJ-34 | pan-PARP | |
| PKR inhibitor | PKR | - |
| PP1 (analog)** | Src, Fyn, Lck | |
| PP1 analog** | Src | |
| PP2 | Lck | |
| PRIMA-1 | p53 activator | - |
| Purvalanol A** | CDK2 | |
| purvalanol A** | CDK | |
| R59022 | DAG kinase | |
| radicicol | Hsp90 | - |
| Radicicol | HSP90 | |
| RAF1 kinase inhibitor I | Raf | |
| Rapamycin | p70 S6K | |
| RHC80267 | DAG lipase | |
| Ro 5-4864 | MPTP | |
| Ro-20-1724 | PDE (cAMP) | |
| Rotenone | mitochondrial complex I | - |
| Rp-8-CPT-cGMPS** | PKG | |
| Rp-8-CPT-cGMPS** | PKG | |
| RS 102895 | CCR2 | - |
| Ruxolitinib | JAK | |
| S2101 (LSD1 inhibitor II) | LSD1 | |

| | | |
|---|---|---|
| Sanguinarine | Na/K/Mg ATPase | - |
| SB 203580 | p38 (MAPK) | |
| SB 218078 | Chk 1 | |
| SB 225002 | CXCR2 | |
| SB 328437 | CCR3 | - |
| SB 431542 | TGF-b receptor | |
| SB202190 | p38 | |
| SB218078 | Chk | |
| SB239063 | p38 | |
| SB431542 | TGF-βRI | |
| Scriptaid | HDAC | + |
| SD208 | TGFb-R | |
| SIRT1 inhibitor III | SIRT1 | |
| Sodium salicylate | COX | |
| sorafenib | Multi-kinases | - |
| SP600125** | JNK | |
| SP600125** | JNK | |
| Staurosporine | PKC, PKA, PKG, MLCK | |
| SU11274 | Met | |
| SU11652 | PDGFR | |
| SU1498** | Flk-1 | |
| SU1498** | VEGFR | |
| SU4984 | FGFR | |
| SU5402 | FGFR | |
| SU6656 | Fyn | |
| Sulindac sulfide | COX-1 | |
| sunitinib malate | Multi-kinases | - |
| Swainsonine | a-mannosidase | |
| Syk inhibitor | Syk | |
| t-Butylhydroquinone (BHQ) | Ca-ATPase | |
| tamibarotene | retinoids | |
| Tamoxifen, citrate | antitumor (ER) | |
| TBB** | CKII | + |
| TBB** | CK | |
| temozolomide | DNA alkylation | |
| temsirolimus | mTOR | - |
| Tenovin-6 | SIRT1/2 | |
| Terreic acid** | Burton's tyrosine kinase(BTK) | |
| Terreic acid** | BTK | |
| TG003 | Clk | |
| TGF-b RI kinase inhibitor II | TGF-βRI | |
| thalidomide | thalidomide family | |
| Thapsigargin | Ca-ATPase | - |
| Theophylline | cyclicphosphodiesterase | + |
| Thiazovivin | ROCK | |
| TMPyP4 | Telomerase | |
| TOFA | acetyl-CoA carboxylase (ACC) | - |
| Torkinib | mTOR | |
| Tpl2 kinase inhibitor | Tpl2 | |
| tretinoin | retinoids | |
| Trichostatin A | HDAC | |
| TrkA inhibitor | TrKA | |
| Troglitazone | PPAR-g activator | |
| Tunicamycin | glycosylation | |
| TWS119 | GSK-3 | |
| TX-1918 | eEF2 | |
| U-0126** | MEK | |
| U0126** | MEK | |
| UNC0638 | G9a | |
| Valeryl salicylate | COX-1 | |
| Valinomycin | K ionophore | |
| Vandetanib | Multi-kinases | |
| VEGF recptor 2 kinase inhibitor I | VEGFR | |
| VEGFR receptor tyrosine kinase inhibitor II | VEGFR | |
| Vemurafenib | BRAF | |
| Verapamil | Ca channel, MDR | |
| Vinblastine sulfate | antitumor (tubulin) | |
| Vismodegib | Hedgehog | |
| vorinostat | HDAC | - |
| Wortmannin** | PI3K | |
| Wortmannin** | PI3K | |

| | | |
|---|---|---|
| WP1066 | STAT3 | - |
| Xanthohumol | DAG acyltransferase (DGAT) | |
| XAV939 | tankyrase-selective PARP | |
| Y-27632 | ROCK | |
| Y27632 | ROCK | |
| YM155 | Survivin | - |
| Z-GLF-CMK | cathepsin G | - |
| Z-VAD-FMK | caspase | |
| Zaprinast | PDE (cGMP) | |
| ZM 336372 | Raf | |

*fluorescence intensity of GFP-expressing F. novicida >5000 higher than DMSO control was determined as positively regulating inhibitors (+), and >4000 lower than the control was determined as negatively regulating inhibitors (-).

**Same compounds, but derived from different providers.
