# Supplementary material for: Role of the JAK2/STAT3 pathway on infection of Francisella novicida
Source: PLoS One. 2024 Sep 10;19(9):e0310120. doi: 10.1371/journal.pone.0310120 (PMC11386456; doi:10.1371/journal.pone.0310120)
Supplement: Attachment — Submitted filename: Response to Reviewers.docx [file pone.0310120.s003.docx]

Thank you for reviewing our paper entitled '' Effect of the JAK2/STAT3 Pathway on Internalization of Francisella novicida'' by Matsumoto *et al*. (PONE-D-24-27526), and giving us an opportunity to submit our manuscript again*.*

We corrected our manuscript according to comments of the editor and reviewers. We believe that the revised manuscript is improved significantly with the referees’ advice and now acceptable for publication in *PLOS One*.

1. When submitting your revision, we need you to address these additional requirements. Please ensure that your manuscript meets PLOS ONE's style requirements, including those for file naming. The PLOS ONE style templates can be found at

https://journals.plos.org/plosone/s/file?id=wjVg/PLOSOne_formatting_sample_main_body.pdf and

https://journals.plos.org/plosone/s/file?id=ba62/PLOSOne_formatting_sample_title_authors_affiliations.pdf

--As you suggested, we checked the requirements and made small changes, e.g. to font sizes.

2. We noticed you have some minor occurrence of overlapping text with the following previous publication(s), which needs to be addressed:

https://www.frontiersin.org/journals/cellular-and-infection-microbiology/articles/10.3389/fcimb.2022.1027424/full

In your revision ensure you cite all your sources (including your own works), and quote or rephrase any duplicated text outside the methods section. Further consideration is dependent on these concerns being addressed.

-- As you suggest, we checked our manuscript using iThenticate software and rephrase some sentences (Line 23-24, 92-93, 162-163).

3. We note that the grant information you provided in the ‘Funding Information’ and ‘Financial Disclosure’ sections do not match.

When you resubmit, please ensure that you provide the correct grant numbers for the awards you received for your study in the ‘Funding Information’ section.

-- As you suggest, we arranged the ‘Funding Information’.

4. Thank you for stating the following financial disclosure: "SM: JST SPRING Grant Number JPMJSP2111 TS: JSPS KAKENHI Grant Number 22K07054, MW: JSPS KAKENHI Grant Number 21H02360".

Please state what role the funders took in the study. If the funders had no role, please state: "The funders had no role in study design, data collection and analysis, decision to publish, or preparation of the manuscript." If this statement is not correct you must amend it as needed.

Please include this amended Role of Funder statement in your cover letter; we will change the online submission form on your behalf.

--As you suggested, we added the sentence in cover letter.

5. Thank you for stating the following in the Acknowledgments Section of your manuscript: "This work was supported by JST SPRING Grant Number JPMJSP2111, JSPS KAKENHI Grant Number 22K07054, JSPS KAKENHI Grant Number 21H02360, and Molecular Profiling Committee, Grant-in-Aid for Transformative Research Areas “Advanced Animal Model Support (AdAMS)” from JSPS KAKENHI Grant Number JP 22H04922."

We note that you have provided funding information that is not currently declared in your Funding Statement. However, funding information should not appear in the Acknowledgments section or other areas of your manuscript. We will only publish funding information present in the Funding Statement section of the online submission form.

Please remove any funding-related text from the manuscript and let us know how you would like to update your Funding Statement. Currently, your Funding Statement reads as follows: "SM: JST SPRING Grant Number JPMJSP2111

TS: JSPS KAKENHI Grant Number 22K07054, MW: JSPS KAKENHI Grant Number 21H02360".

Please include your amended statements within your cover letter; we will change the online submission form on your behalf.

--Thank you for checking our funding information. We removed Acknowledge section from the manuscript. The information listed above is correct. We added explanation about this information in cover letter.

6. Please review your reference list to ensure that it is complete and correct. If you have cited papers that have been retracted, please include the rationale for doing so in the manuscript text, or remove these references and replace them with relevant current references. Any changes to the reference list should be mentioned in the rebuttal letter that accompanies your revised manuscript. If you need to cite a retracted article, indicate the article’s retracted status in the References list and also include a citation and full reference for the retraction notice.

--We checked references and found no problems.

Reviewers' comments:

Reviewer's Responses to Questions

Comments to the Author

Reviewer #1: The paper entitled Effect of the JAK2/STAT3 Pathway on Internalization of Francisella novicida; is very interning. It was written well and provided with sufficient data. However, some concern need to be addressed.

--Thank you for your reviewing. As you suggested we corrected our manuscript.

1- Why the authors choose Cucurbitacin I? What about the other inhibitors?

--In this study, we identified 56 inhibitors that negatively regulate F. novicida infection. Among them 3 inhibitors were related to Jak-2/STAT3 pathway. Therefore we thought that Jak-2/STAT3 pathway might be involved in F. novicida infection. Cucurbitacin was used as a representative inhibitor of the Jak-2/Stat3 pathway. Additionally, other promising inhibitors have been identified, including those targeting mTOR and p53, which are currently undergoing analysis. To explain this we rewrote sentences (line 198-202).

2- What is the source of Cucurbitacin I?

--Cucurbitacin I is a natural cell-permeable triterpenoid isolated from Cucurbitaceae, and is a selective inhibitor of JAK2/STAT3. The Cucurbitacin I is explained in Discussion section (line334-335).

3- What is the purpose of providing merge, DIC ,and GFP?

--DIC was used to show the location of the cells; the intention was to show that the bacteria were intracellularly located by using merged images with the DIC.

4- Interfiering of the JAK2/STAT3 Pathway by Cucurbitacin, how it affect the phenotype of the bacteria?

--We thought cucurbitacin might have an antibiotic effect, so we added cucurbitacin to the medium and investigated growth, but found that cucurbitacin had no effect on bacterial growth (Fig 2). From these data we consider that cucurbitacin does not affect the phenotype of *F. novicida*.

5- The title of the paper is not in line with the context. It should be revised by the authors. Internalization, and withing the text is all about inhibiting the pathway by Cucurbitacin I.

--We changed the title as you suggested (line 1).

Reviewer #2: The paper was checked as Effect of the JAK2/STAT3 Pathway on Internalization of Francisella novicida. It is an exciting paper on the plant extract main ingredient effect on the bacteria, which bring valuable insight into the bacterial physiology and biology within the cells.

--Thank you for your review. We corrected our manuscript as you suggested.

However, the authors must explain how they choose Cucurbitacin I, and what was the rate of inhibition?

--In this study, we identified 56 inhibitors that negatively regulate F. novicida infection. Among them 3 inhibitors were related to Jak-2/STAT3 pathway. Therefore we thought that Jak-2/STAT3 pathway might be involved in F. novicida infection. Cucurbitacin was used as a representative inhibitor of the Jak-2/Stat3 pathway. Additionally, other promising inhibitors have been identified, including those targeting mTOR and p53, which are currently undergoing analysis. To explain this we rewrote sentences (line 198-202). In the first screening we used the intensity of GFP, so the rate of inhibition could only be expressed as a relative value compared to the control (table S1). Therefor, we measured the effect of Cucurbitacin I on *F. novicida* infection in Fig. 1.

The title of the paper also confusing as only bacteria and the pathway included, but not the effect of plant extract on the pathway.

--We changed the title as you suggested (line 1).

Overall, the manuscript is well written, and I would recommend to be published in PLoS One after a minor revision.

While revising your submission, please upload your figure files to the Preflight Analysis and Conversion Engine (PACE) digital diagnostic tool, https://pacev2.apexcovantage.com/. PACE helps ensure that figures meet PLOS requirements. To use PACE, you must first register as a user. Registration is free. Then, login and navigate to the UPLOAD tab, where you will find detailed instructions on how to use the tool. If you encounter any issues or have any questions when using PACE, please email PLOS at figures@plos.org. Please note that Supporting Information files do not need this step.

--We checked figures with PACE and converted tiff files were uploaded to the journal online system.
